# Hydrogen Bonding in a *l*-Glutamine-Based Polyamidoamino Acid and its pH-Dependent Self-Ordered Coil Conformation

**DOI:** 10.3390/polym12040881

**Published:** 2020-04-10

**Authors:** Federica Lazzari, Amedea Manfredi, Jenny Alongi, Fabio Ganazzoli, Francesca Vasile, Giuseppina Raffaini, Paolo Ferruti, Elisabetta Ranucci

**Affiliations:** 1 Dipartimento di Chimica, Università degli Studi di Milano, via C. Golgi 19, 20133 Milano, Italy; federica.lazzari@unimi.it (F.L.); amedea.manfredi@unimi.it (A.M.); jenny.alongi@unimi.it (J.A.); francesca.vasile@unimi.it (F.V.); 2 Dipartimento di Chimica, Materiali ed Ingegneria Chimica “G. Natta” Politecnico di Milano, Piazza Leonardo da Vinci 32, 20131 Milano, Italy; fabio.ganazzoli@polimi.it

**Keywords:** polyamidoamino acid, *l*-glutamine, chiral polymers, hydrogen bonding, molecular dynamics, diffusion ordered NMR spectroscopy, NOESY

## Abstract

This paper reports on synthesis, acid–base properties, and self-structuring in water of a chiral polyamidoamino acid, M-*l*-Gln, obtained from the polyaddition of *N,N′*-methylenebisacrylamide with *l*-glutamine, with the potential of establishing hydrogen bonds through its *prim*-amide pendants. The M-*l*-Gln showed pH-responsive circular dichroism spectra, revealing ordered conformations. Structuring was nearly insensitive to ionic strength but sensitive to denaturing agents. The NMR diffusion studies were consistent with a population of unimolecular nanoparticles thus excluding aggregation. The M-*l*-Gln had the highest molecular weight and hydrodynamic radius among all polyamidoamino acids described. Possibly, transient hydrogen bonds between *l*-glutamine molecules and M-*l*-Gln growing chains facilitated the polyaddition reaction. Theoretical modeling showed that M-*l*-Gln assumed pH-dependent self-ordered coil conformations with main chain transoid arrangements reminiscent of the protein hairpin motif owing to intramolecular dipole moments and hydrogen bonds. The latter were most numerous at the isoelectric point (pH 4.5), where they mainly involved even topologically distant main chain amide N–H and side chain amide C=O brought to proximity by structuring. Hydrogen bonds at pH 4.5 were also suggested by variable temperature NMR. The 2D NOESY experiments at pH 4.5 confirmed the formation of compact structures through the analysis of the main chain/side chain hydrogen contacts, in line with MD simulations.

## 1. Introduction

**Scheme 1:**
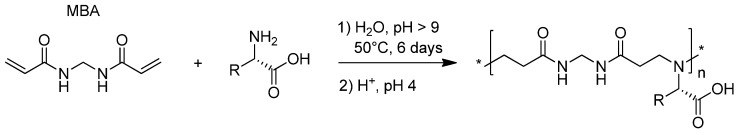
Synthesis of polyamidoamino acids (PAACs).

Many natural polymers spontaneously self-organize into stable and ordered conformations and assemble into supramolecular structures by means of non-covalent interactions [1,2,3]. Chirality, [4,5,6] hydrophobic domains [7,8,9], and hydrogen bonds [9,10,11] play a relevant role in inducing formation of specific three-dimensional structures. Chiral synthetic and hybrid polymers may also exhibit structuring ability [12,13,14,15] and open interesting perspectives for applications such as catalysis [16,17,18,19], drug-delivery [20,21], chiral recognition [22,23], chiral resolution [24,25,26], biosensing [27,28,29], and bioimaging [30]. Recently, the chiral representatives of a new polymer family deriving from natural α-amino acids, polyamidoamino acids (PAACs), have been shown to assume stable conformations in water [31,32,33,34]. Polyamidoamino acids are obtained by the step-wise Michael polyaddition of natural α-amino acids or their stereoisomers with *N,N*′-methylenebisacrylamide (MBA) (Scheme 1).

The peculiarity of PAACs, compared to other natural and synthetic polymers prepared from or inspired by α-amino acids, for instance, polypeptides, polypeptoids, and poly-*N*-acrylamido acids, consists in the retention, in addition to the chirality, of the amphoteric properties of the starting α-amino acids. In fact, the polyaddition reaction with MBA converts the *prim*-amine group of the α-amino acid into a still basic *tert*-amine group and does not involve the carboxyl group. 

The PAACs studied so far have been classified based on the structural features of the α-amino acid side substituents, namely, basic PAACs, obtained from *l*-, *d*- or *d,l*-arginine (called ARGO7 stereoisomers) [31,32] and alkyl-substituted PAACs, obtained from *l*-alanine (M-*l*-Ala), *l*-valine (M-*l*-Val), and *l*-leucine (M-*l*-Leu) [33]. In addition, homo- and copolymeric *l*-tryptophan-based PAACs were studied [34]. 

Circular dichroism (CD) spectroscopy showed that all chiral PAACs self-structured in water with a pH-dependent molar ellipticity. Conformational changes were rapid, reversible, and modestly influenced by temperature, molecular weight, and ionic strength, whereas the effect of denaturing agents, such as urea and guanidinium chloride, depended on the structure of the α-amino acid used as monomer. Molecular dynamics studies on *l*-ARGO7, M-*l*-Ala, M-*l*-Val, and M-*l*-Leu [32,33], carried out on decamers as models of the higher molecular weight samples actually adopted in experimental studies, revealed that they all assumed in water compact structures with gyration radii, *R_g_*, in the range 0.75–1.11 nm. The PAAC main chains organized in transoid arrangements with hairpin-like conformations stabilized by intramolecular interactions, that is, local dipoles and few H-bonds between either main chain groups or side groups or both. The chiral carbon atoms of the α-amino acid moieties adjacent to the main chain induced structuring, irrespective of the nature of the α-amino acid side group [32,33]. In spite of their compactness, efficient hydration was allowed by the cavities revealed inside the coil by theoretical modeling. 

Based on this premise, it was interesting to study the solution properties of a PAAC whose repeat unit carried a side substituent capable of giving rise to stable H-bonds. To this purpose, the α-amino acid of choice was *l*-glutamine, thanks to its recognized ability to associate even in dilute solution and to the role played by its units in promoting protein–protein associations [35,36]. A PAAC, M-*l*-Gln, was then prepared by the polyaddition of *l*-glutamine with MBA, and its solution properties were compared with those of the previously described PAACs. The purpose of this paper was to report on the results obtained.

## 2. Materials and Methods

### 2.1. Materials

Solvents and reagents, unless otherwise indicated, were analytical grade commercial products and used as received. *l*-Glutamine (≥99.5%) was purchased from Sigma–Aldrich (Milano, Italy). *N,N*′-Methylenebisacrylamide (MBA, 96%) was purchased from Acros Organics (Milano, Italy) and lithium hydroxide monohydrate (≥98%) was supplied by Honeywell Fluka (Steinheim, Westphalia, Germany). Hydrochloric acid and sodium hydroxide volumetric standard solutions were purchased from Fluka analytics (Milano, Italy). Ultrapure water (18 MΩ·cm^−1^), produced with a Millipore Milli-Q^®^ apparatus (Darmstadt, Hesse, Germany), was used to prepare solutions.

### 2.2. Characterizations

1D-^1^H-, 1D-^13^C- and ^1^H,^13^C-HSQC NMR spectra were obtained in 9:1 H_2_O:D_2_O and D_2_O at pH 4.5 and 298 K using a Brüker Avance 400 MHz and a Brüker Avance III 400 MHz. Variable temperature NMR (VT-NMR) spectra were recorded at pH 4.5 in the range 298–338 K using a Brüker Avance III 400 MHz. Nuclear overhauser effect spectroscopy (NOESY) experiments with mixing times of 200 and 700 ms were used to evaluate the spatial correlations. In the 1D-^1^H and 2D-NOESY experiments, solvent suppression was achieved by using the excitation sculpting pulse sequence. Diffusion ordered spectroscopy (DOSY) spectra were recorded in D_2_O at pH 4.5 using a Brüker Avance 600 MHz, following the standard Brüker sequence with pre-saturation during relaxation delay for water suppression. 

Size exclusion chromatography (SEC) traces were obtained for all polymers with Toso-Haas TSK-gel G4000 PW and TSK-gel G3000 PW columns connected in series, using a Waters model 515 HPLC pump (Milano, Italy) equipped with a Knauer autosampler 3800 (Knauer, Bologna, Italy), a light scattering (670 nm), a viscometer Viscotek 270 dual detector (Malvern, Roma, Italy), and a refractive index detector (Model 2410, Waters, Milano, Italy). The mobile phase was a 0.1 M Tris buffer (pH 8.00 ± 0.05) solution with 0.2 M sodium chloride. Sample concentration: 20 mg mL^−1^; flow rate: 1 mL min^−1^; injection volume: 20 µL; loop size: 20 µL; column dimensions: 300 × 7.5 mm^2^. The instrument optical constants were determined using PEO 24 kDa as a narrow standard. Before analysis, each sample was filtered through a 0.2 µm Whatman^TM^ syringe filter (Maidstone, UK). 

Dynamic light scattering (DLS) analyses were carried out on 1 mg mL^−1^ polymer solutions prepared in ultrapure water, using a Malvern Zetasizer NanoZS instrument (Malvern, Roma, Italy), equipped with a 532 nm laser fixed 173° scattering angle. Before analysis, each sample was filtered through a 0.2 µm Whatman^TM^ syringe filter (Maidstone, UK). The solution pH was adjusted using 0.1 M HCl or 0.1 M NaOH aqueous solutions. The reported values were averaged over measurements obtained with three different samples. For each sample, the value considered was the average of 10 runs. 

Circular dichroism (CD) spectra were obtained using a JASCO J-500CD spectrometer (Jasco Europe S.r.l., Lecco, Italy) in the range 200–300 nm in a 1 cm path-length quartz cell at 50 nm min^−1^ scan speed. Polymer solutions (0.5 mg mL^−1^) were prepared in 0.1 M NaCl and their pH adjusted with 0.1 M HCl or 0.1 M NaOH using a combined Metrohm microelectrode (Varese, Italy). The CD spectra were normalized based on the molar concentration of the repeat units (1.66 mM) and reported as molar ellipticity (mdeg M^−1^ cm^−1^) versus wavelength. The spectra reported were the average of three runs.

Potentiometric titrations were performed at 25 °C employing a Primatrode Metrohm electrode combined with an NTC temperature sensor and connected to an 827 pH lab Metrohm pH meter (Varese, Italy). A three-point calibration was carried out using standard buffers. The samples were dissolved in 0.1 M NaCl (10 mL) obtaining 0.05 M repeat unit concentrations; the solutions were deaerated by ultrapure nitrogen purging, then the pH was adjusted to 1.2–1.3 with 1 M HCl (0.7 mL) and the resultant solutions titrated with 0.1 M NaOH. All titrations were performed in quadruplicate.

### 2.3. Synthesis of M-l-Gln

*l*-Glutamine (3.67 g, 24.99 mmol), MBA (4.02 g, 25.00 mmol), and lithium hydroxide monohydrate (1.07 g, 24.99 mmol) were added to water (8.5 mL). The resultant slurry was heated to 50 °C and magnetically stirred till complete dissolution of solids. Stirring was then discontinued and the reaction mixture maintained at 50 °C for 6 days with occasional shaking. After this time, the reaction mixture was acidified to pH 4 with 6 M HCl and ultrafiltered through a membrane with a nominal molecular weight cut-off of 100,000 to eliminate possible particulate impurities. The passed-through portion was then further ultrafiltered three times through a membrane with a nominal cut-off of 5000, each time diluting to the maximum cell volume for a total water passage of of 1000 mL. The product was retrieved as an off-white powder by freeze-drying the retained portion (yield 72%).

^1^H-, ^13^C-NMR, and ^1^H,^13^C-HSQC spectra and assignments are reported in Supplementary Materials Figures S1–S3 and Table S1.

Preparations and analyses were performed in triplicate, always giving consistent results.

### 2.4. Molecular Mechanics (MM) and Molecular Dynamics (MD) Simulations 

Molecular mechanics (MM) and molecular dynamics (MD) simulation protocols [32,33] were applied using the InsightII/Discover packages [37] and adopting the consistent valence force field (CVFF). The starting configuration of a model M-*l*-Gln decamer was obtained using the templates in the Builder module. For simulations both in the dielectric medium, using a distance dependent dielectric constant, and in explicit water, all energy minimizations were carried out up to an energy gradient < 4 × 10^−3^ kJ mol^−1^ Å^−1^. The MD simulations were performed at a constant temperature (300 K), controlled through the Berendsen thermostat. The integration of the dynamical equations was carried out with the Verlet algorithm using a time step of 1 fs, and the instantaneous coordinates were periodically saved for further analysis. In implicit solvent, the length of the MD runs was 5 ns and the subsequent runs in explicit water lasted for additional 500 ps for the three simulation environments considered, pH 1.0, 4.5, and 12.0. In explicit water, a 44 Å cubic cell was considered, containing one M-*l*-Gln decamer and approximately 2700 water molecules with periodic boundary conditions.

During the MD runs, the conformational changes were monitored by considering the root mean square distance (RMSD) [38,39] among the different frames saved periodically. In addition, the changes on time of the total and potential energy and its components were monitored. In all cases, these energy components showed an initial decrease and then fluctuated around a constant value, indicating achievement of a state of equilibrium.

## 3. Results and Discussion

### 3.1. Synthesis of M-l-Gln

**Scheme 2:**
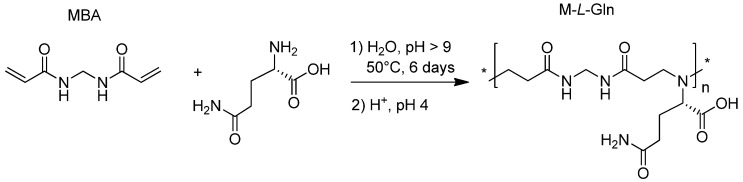
Synthesis of M-*l*-Gln.

The M-*l*-Gln was prepared by the polyaddition of *l*-glutamine with MBA according to Scheme 2. The reaction involved exclusively the α-amino acid amine group as ascertained by ^1^H-, ^13^C- and ^1^H,^13^C-HSQC NMR spectroscopy (Supplementary Materials Figures S1–S3 and Table S1).

The reaction conditions were essentially the same as for ARGO7 [31,32], M-*l*-Ala, M-*l*-Val, and M-*l*-Leu [33]. The molecular weight of M-*l*-Gln, determined by size exclusion chromatography (SEC), was significantly higher than those of all previously described PAACs and also polydispersity was higher (Table 1). The broad polydispersity is in line with the relative abundance of terminal groups revealed by NMR. The presence of either very short oligomers or monomer impurities was not detected by diffusional NMR experiments (Section 3.6). In the ^1^H-, ^13^C-, and HSQC NMR spectra, several peaks have been detected that may be ascribed to segments with reduced rotational freedom. In particular, in the ^1^H-NMR spectrum, the peak at ~4.0 ppm, attributed to the C-H α to the COOH groups of glutamine units, is probably due to the branches originated by the participation of some amidic NH_2_ of the glutamine pendants in the aza-Michael polyaddition (see Supplementary Materials Figure S1b). Some examples of the aza-Michael addition of *prim*- or *sec*-amides in the presence of bases are reported [40,41]. In the case of M-*l*-Gln, the amide NH_2_ addition gives rise to branches and to a corresponding number of terminals. In the meantime, this provides a reasonable explanation of the unusually high molecular weight and polydispersity of M-*l*-Gln compared to all other PAACs.

### 3.2. Acid–Base Properties of M-l-Gln

The M-*l*-Gln contains one carboxyl- and one *tert*-amine group per repeat unit and, therefore, is an amphoteric polyelectrolyte. As such, its *pK_a1_* (carboxyl group) and *pK_a2_* (*tert*-amine group) depend on the dissociation degree *α*, according to the modified Henderson–Hasselbalch equation (Equation (1))

(1) $$pH=pK_{a}-\beta \;log\frac{1-\alpha }{\alpha }$$

where *K_a_* is the “apparent” weak acid dissociation constant of the group being pH-determining in the buffer titration zone considered and *β* is the Katchalsky and Spitnik parameter [42] accounting for possible interactions among ionizable groups. *pK_a1_* and *pK_a2_* were determined from the two half-equivalence points of the potentiometric titration, for both of which *pH = pK_a_*; *β_1_* and *β_2_* were determined by the procedures described in the Supplementary Materials (see also Figure S4). The *pK_a_* and *β* values of M-*l*-Gln (Table 2) were in line with those previously obtained for PAACs prepared from alkyl substituted α-amino acids [33]. 

**Figure 1:**
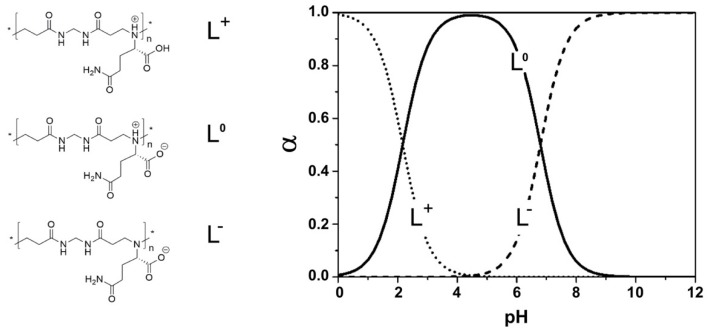
Ionization states and speciation diagram of M-*l*-Gln.

The repeat units of M-*l*-Gln can exist in three ionization states: positively charged (L^+^), electroneutral (L^0^), and negatively charged (L^−^) (Figure 1). The pH-dependent speciation curves (Figure 1) were determined from the *pK_a_* and *β* values reported in Table 2 by the method described in the Supplementary Materials (see also Figure S4).

### 3.3. Dynamic Light Scattering (DLS) Measurements

**Figure 2:**
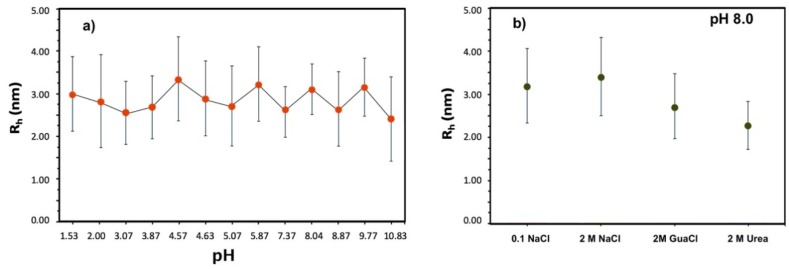
Dependence of M-*l*-Gln hydrodynamic radius, *R_h_*, on: (**a**) pH (in 0.1 M NaCl); (**b**) ionic strength (in 2 M NaCl) and denaturing agents (guanidinium chloride, GuaCl, and urea). Data obtained by DLS at 25 °C on 1 mg mL^−1^ solutions.

The pH-dependence of M-*l*-Gln hydrodynamic radius, *R_h_*, was studied by DLS measurements carried out on 1 mg mL^−1^ solutions in 0.1 M NaCl. It turned out that *R_h_* did not significantly change with pH (Figure 2a). The values obtained were higher than those of all previously described PAACs under the same conditions, in agreement with its higher molecular weight [32,33]. At pH 8.0, the *R_h_* values did not significantly change in either 2 M NaCl or 2 M guanidinium chloride (GuaCl) or 2 M urea as potential denaturing agents (Figure 2b). 

### 3.4. Circular Dichroism Spectrocopy

**Figure 3:**
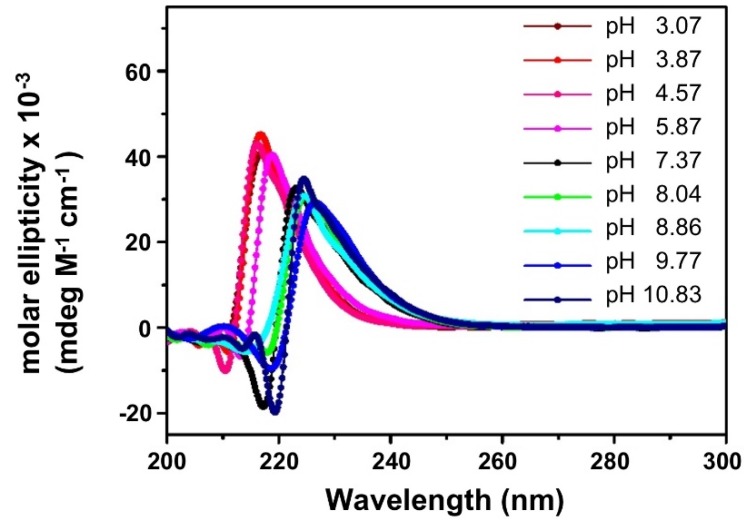
pH-Dependence of M-*l*-Gln CD spectra. Data obtained at 25 °C in 0.1 M NaCl with a repeat unit concentration of 1.66 mM.

The circular dichroism (CD) spectra of M-*l*-Gln in 0.1 M NaCl and in the pH range 3–11 demonstrated the presence of self-structuring (Figure 3). Like all PAACs [31,32,33,34], M-*l*-Gln showed pH-dependent CD patterns due to the conformational changes induced by the changes in the ionization state. However, unlike other PAACs, the M-*l*-Gln spectra obtained at acidic pH exhibited positive molar ellipticity with maxima centered at 217 nm and only modest negative peaks with minima centered at 210–215 nm. These positive peaks were mainly attributed to the weak n → π* transition of the CONH groups [43] present both in the polymer backbone and in the *l*-glutamine side group. Furthermore, by increasing pH, the molar ellipticity maxima underwent a progressive bathochromic shift, up to 227 nm at pH 9.77, with reduced intensities. 

**Figure 4:**
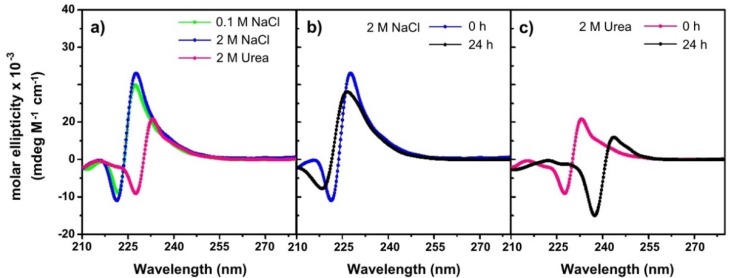
M-*l*-Gln CD spectra dependence on: ionic strength (0.1 M and 2 M NaCl), panels (**a**) and (**b**); denaturing agent (urea), panels (**a**) and (**c**). Data obtained at 25 °C, pH 8.0 in 0.1 M NaCl with a repeat unit concentration of 1.66 mM.

The dependence of CD spectra on ionic strength and denaturing agents at pH 8.0 was investigated at zero time and after 24 h. The spectral pattern was nearly unaffected by increasing the ionic strength up to 2 M NaCl (Figure 4a), remaining unchanged over time (Figure 4b). By contrast, in 2 M urea, both negative and positive peaks underwent significant bathochromic shifts with maximum displacement to 237 and 243 nm, respectively (Figure 4a). Correspondingly, the intensity of the positive peak decreased, whereas that of the negative peak increased. These spectral variations were more pronounced after 24 h (Figure 4c), suggesting a progressive breakdown of hydrogen bonds.

### 3.5. Molecular Simulation Studies

Room temperature molecular dynamics (MD) simulations with final energy minimization using molecular mechanics (MM) methods were carried out on M-*l*-Gln decamer in three ionization states, that is, with positive (L^+^, pH 1.0), null (L^0^, pH 4.5), and negative (L^−^, pH 12.0) charge per repeat unit (Figure 1), adopting the same protocol, software and force field used in previous theoretical studies on *l*-ARGO7 [32], M-*l*-Ala, M-*l*-Val, and M-*l*-Leu [33]. Starting from a fully elongated chain, M-*l*-Gln conformational properties were studied in an effective medium using a distance-dependent dielectric constant, followed by further simulation runs in explicit water adopting a periodic simulation box. In water, after a preliminary energy minimization at 300 K, the MD runs were performed up to the equilibrium state, monitoring both the conformational changes using the RMSD among the conformations saved during MD run and the time dependence of the energy components. 

**Figure 5:**
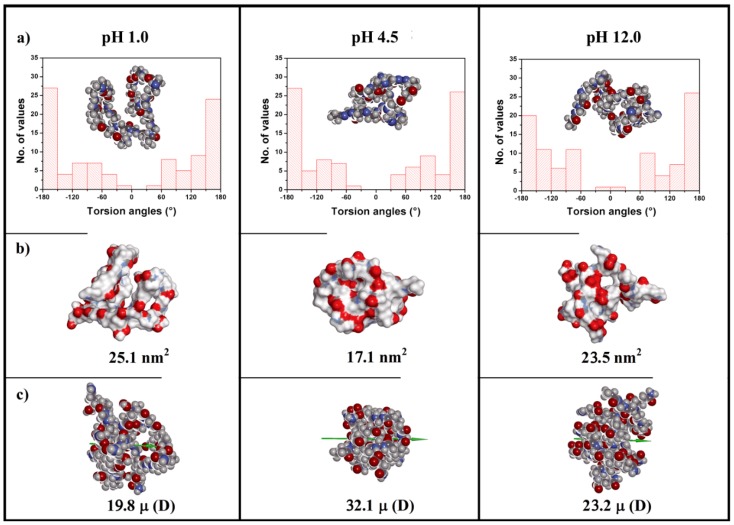
(**a**) Main chain molecular conformations in water at the end of the MD runs for M-*l*-Gln at pH 1.0, 4.5, and 12.0 and torsion angle distributions around the main chain bonds: the histogram are shown with a binning of 300 K. C atoms: dark grey; H atoms: light grey; N atoms: blue; O atoms: red. (**b**) Solvent accessible surface area in water. Surface area near C atoms: dark grey; near H atoms: light grey, near N atoms: blue; and near O atoms red. (**c**) Dipole moments in water. Color codes are the same as in panel (**a**).

The MD runs in the effective dielectric medium led to compact coiled conformations with only minor swelling after explicit hydration in all three simulation environments, particularly in the case of the electroneutral (L^0^, pH 4.5) and anionic (L^−^, pH 12.0) states. The final M-*l*-Gln conformations in the three optimized geometries achieved after the MD runs in explicit water are shown in Figure 5.

The intramolecular interactions led to a few folded local structures of the polymer main chain reminding the hairpin conformation, a structural protein motif that may lead to β-sheet conformation (Figure 5a). The hairpin motifs were best seen at pH 1.0, owing to the almost flat geometry locally assumed by large main chain portions. Shorter but more numerous hairpins were found at pH 4.5 and 12.0. As observed for other PAACs [32,33], the M-*l*-Gln decamer in the electroneutral state (L^0^, Table 3) showed the minimum gyration radius, *R_g_*. However, in the cationic and anionic decamers (L^+^ and L^−^), *R_g_* increased only by a 1.2–1.3 factor thus ruling out the hypothesis of extended conformations or large effects induced by the electric charge. The electroneutral decamer also showed the minimum surface area accessible to the solvent, in line with the small *R_g_*, whereas the volume was the largest, due the cavities present inside the structured coil (Table 3). 

This conformational behavior could also be gauged through the distribution of the dihedral angles along the main chain (Figure 5a). As in other PAACs, a large preference was shown by the transoid arrangement of the main chain which lead to a strong preference for torsion angles near ±180°, particularly for the cationic (L^+^) and the electroneutral (L^0^) decamers. In the anionic chains (L^−^), the transoid arrangements were somewhat shorter, while the *gauche* arrangements with torsion angles at ± 60° were more numerous. As a final observation, the fact that the *R_g_* values of the isolated M-*l*-Gln decamer in all three simulation environments were very close to those obtained in the same conditions for *l*-ARGO7 [32], M-*l*-Ala, M-*l*-Val, and M-*l*-Leu decamers [33] indicated that the molecular size was mainly governed by the conformational properties of the main chain, in turn induced by the chirality, and not dependent on the nature of the α-amino acid side groups. 

#### 3.5.1. Molecular Mechanics: Conformations of the Optimized Geometries 

**Figure 6:**
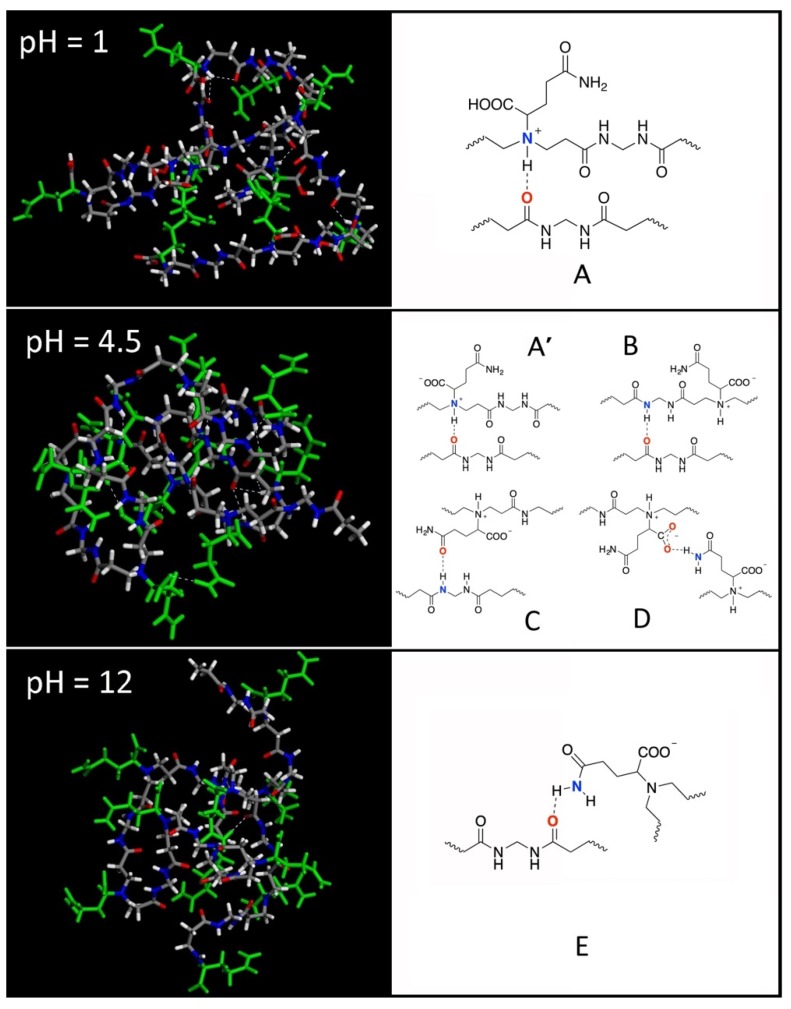
Molecular conformations of M-*l*-Gln at pH 1.0, 4.5 and 12.0, corresponding to the optimized geometries achieved after the MD runs in water. H-bonds are represented by white, dotted lines. All atoms of the *l*-glutamine residues are colored in green, whereas the main chain atoms are colored using the same code as in Figure 5. The structures (**A**), (**A′**). (**B**), (**C**), (**D**) and (**E**) are line drawings showing the corresponding intramolecular H-bond patterns (see text).

The pH-dependence of M-*l*-Gln conformations was elucidated by the optimized geometries obtained at the end of the MD runs in water at different pH values (Figure 6). 

Overall, the M-*l*-Gln decamer in the cationic (L^+^, pH 1.0) and anionic (L^−^, pH 12.0) states featured only a few H-bonds. In particular, in the cationic state, four main chain/main chain H-bonds were present (Figure 6A), whereas in the anionic state, only a single main chain/side chain H-bond was present (Figure 6E). Furthermore, in the latter simulation environment, the main chain atoms were organized in the central part of the coil, whereas all *l*-glutamine side groups protruded outwards hence exposed to the water molecules. Conversely, in the electroneutral state (L^0^, pH 4.5), the *l*-glutamine side groups were also located inside, not only on the external surface of the coil. In addition, twelve H-bonds were found involving topologically distant repeat units, justifying the more compact conformation hence the lower *R_g_* value (Table 3). Of these H-bonds, two were due to the main chain/main chain interactions (Figure 6A′,B), seven to main chain/side chain interactions (Figure 6C), and three to side chain/side chain interactions (Figure 6D). These data indicated that H-bonds involving the main chain amide N-H and the side chain amide C=O were mainly responsible for the stability of the self-structured coiled conformation at pH 4.5. 

#### 3.5.2. Molecular Dynamics: H-Bond Distribution at pH 4.5

**Figure 7:**
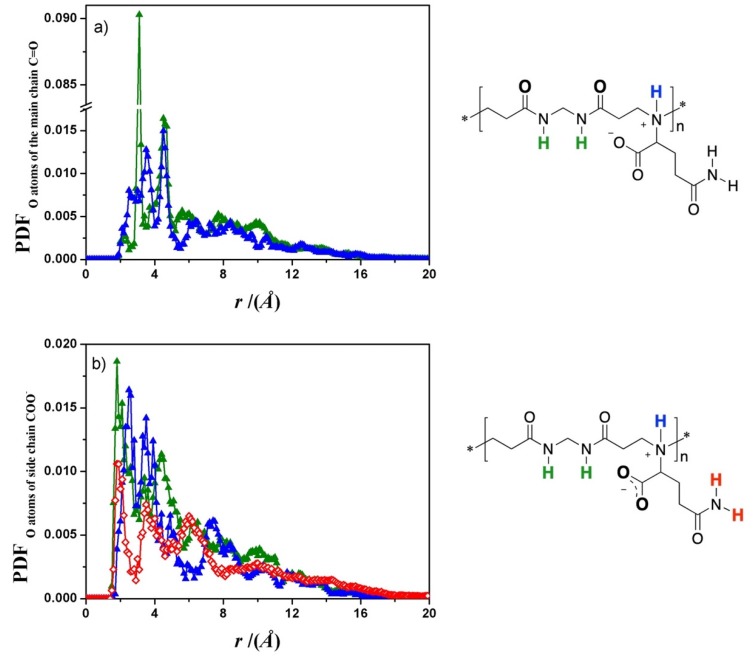
Pair distribution function (PDF) for M-*l*-Gln at pH 4.5. (**a**) Distribution of distances between the oxygen atoms of the main chain C=O: (*i*) the H atoms of the main chain amide groups (green); (*ii*) the H atoms of the protonated *tert*-amine groups (blue). (**b**) Distance distribution between the oxygen atoms of the carboxylate groups and (*i*) the H atoms of the main chain amides (green); (*ii*) the H atoms of the protonated *tert*-amines (blue); (*iii*) the H atoms of the amides in the side groups (red).

To analyze the conformation of the M-*l*-Gln decamer at pH 4.5, in which a large number of intramolecular H-bonds were observed in a rather compact structure, the pair distribution function (PDF), describing the distribution of the distances between pairs of atoms belonging either to the main- or to the side chains, was calculated during the room temperature MD runs in water (Figure 7). As previously found [37,39], the PDF is a convenient tool for describing the conformational properties of molecules in solution and comparing theoretical data with signals in NOESY experiments. Here, for distances < 5 Å, the PDF allowed to describe the formation and stability of the H-bonds dynamically formed during the MD trajectory, while for distances > 5 Å, their shape represented a useful geometric descriptor of possible ordered structures.

The graphs shown in Figure 7a refer to the probability density of finding main chain/main chain H-bonds within an isolated electroneutral M-*l*-Gln decamer. The sharp peak in the distance range 2.9 < *r* < 3.2 Å (green) and the peak centered around 4.5 Å (blue) are due to the topologically close atoms, that is, in the same repeat unit and, therefore, are irrelevant for studying hydrogen bond formation among different repeat units. Conversely, the peaks in the range 1.8 < *r* < 2.5 Å indicate two relevant intramolecular H-bonds between main chain groups belonging to topologically distant repeat units: amide C=O/amide N–H (green) and amide C=O/protonated *tert*-amine N-H (blue). These atoms become spatially close thanks to the main chain flexibility, which allows folding, and the favorable dipolar interactions which stabilize their proximity during the MD trajectory. The sharp peaks in the range 3.7 < *r* < 4.0 Å (green) indicate a large probability density of finding the atoms considered at such distances due to the regular compact main chain arrangement in partially ordered parallel strands. The broad multiple peaks at *r* > 4.0 Å are also attributed to ordered structures remaining quite stable during the MD runs. Finally, it may be noted that the PDF gradually vanish at distances consistent with the size of the modeled M-*l*-Gln decamer. 

The graphs in Figure 7b (full symbols) refer to the probability density of finding main chain/side chain H-bonds. The peak at 1.8 Å (green) correspond to the strong H-bonds between the carboxylate oxygens and the main chain amide N–H of different repeat units. The peak centered at 3.7 Å (blue) correspond to weak H-bonds between the oxygens of the carboxylate groups and the hydrogens of the protonated *tert*-amines at a slightly greater distance in an orderly distribution. Considering the blue symbols, the first peak present in the range 2.5 < *r* < 4.5 Å centered at 3.7 Å corresponds to the weaker H-bonds with the hydrogens of the protonated *tert*-amines at a somewhat larger distance (blue symbols) in an ordered distribution. For both blue and green graphs, the PDF calculated in the distance range 3.3 < *r* < 3.6 Å relate to topologically close atoms, whereas the broad peaks at *r* > 4 Å refer to interactions between topologically distant atoms brought to spatial proximity by the structured coil conformation. 

Finally, the graphs in Figure 7b (red empty symbols) relate to the probability density of finding side chain/side chain H-bonds involving the carboxylate oxygens and the amide N–H of the *l*-glutamine residues. The peak at about 6 Å is due to topologically close atoms belonging to the same *l*-glutamine moiety and is, therefore, useless for the study of M-*l*-Gln conformation. Conversely, the peak centered at about 1.8 Å is due to the strong H-bonds between topologically distant side groups becoming spatially close in the stable coiled structure populated during the MD trajectory in water. A second peak at around 3.5 Å corresponds to the ordered arrangement of the side groups. 

### 3.6. NMR Analyses 

The DOSY [44,45], VT-NMR [46] and NOESY [47,48] (see Materials and Methods, Section 2) were used to study the structural and conformational features of M-*l*-Gln in water at the isoelectric point (pH 4.5). The DOSY measurements (Figure S5) allowed to determine the diffusion coefficient, *D*, for all individual M-*l*-Gln proton resonances (Table S1). The *R_h_* value was calculated from the average diffusion coefficient using the Stokes–Einstein equation [44] (Equation (2)):

(2) $$D=k_{B}T/6\pi \eta R_{h}$$

where *k_B_* is the Boltzmann’s constant, *T* the absolute temperature, *η* the solvent viscosity, and *R_h_* the hydrodynamic radius. The *R_h_* value obtained (3.53 nm) was in reasonable agreement with that obtained under the same conditions by DLS measurements (3.25 nm, Figure 2). Both values were well above the 0.75–1.75 nm range typical, at the same pH, of all the other PAACs [32,33] and in line with the exceptionally high M-*l*-Gln molecular weight. 

The presence of H-bonds was verified by VT-NMR (Figure S6), monitoring the amide protons. Indeed, the temperature dependence of their chemical shift, ∆δNH/∆T, is an indicator of the extent of hydrogen bonding. The M-*l*-Gln contains two types of amide groups per repeat unit, the main chain *sec*-amide and the side chain *prim*-amide groups. The chemical shift of amide N–H not involved in H-bonds generally shows significant temperature dependence, that is, −7 ppb K^−1^ [49]. As for M-*l*-Gln, the ∆δNH/∆T of the main chain N-H, −6.0 ppb K^−1^, and that of the side chain N–H, −4.5 ppb K^−1^, were indicative of H-bond formation, in agreement with the results of MM (Section 3.5.1). 

**Figure 8:**
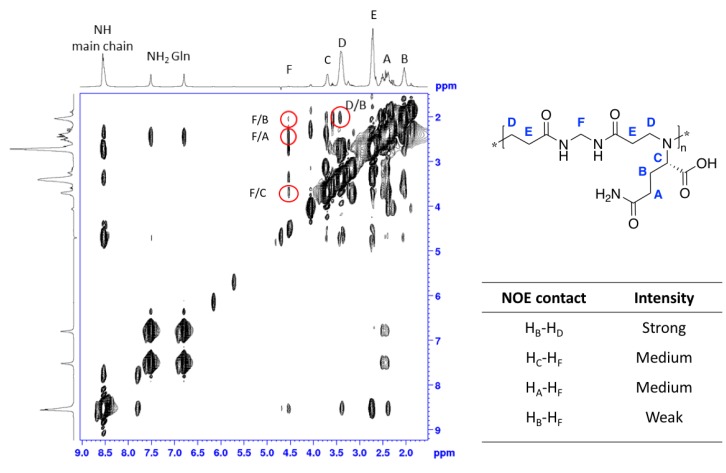
2D-NOESY NMR spectrum of M-*l*-Gln recorded in 9:1 H_2_O:D_2_O at pH 4.5 and 298 K, using a Brüker Avance III 400MHz instrument.

**Figure 9:**
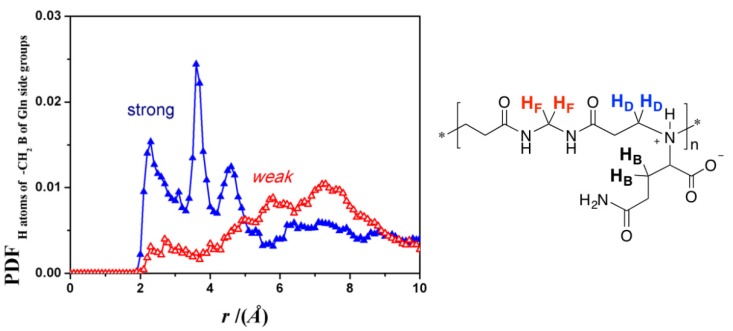
Pair distribution function (PDF) for M-*l*-Gln at pH 4.5. Distribution of distances between the side chain H_B_ atoms (black in the structure of the repeat unit) and the H_D_ (blue) and H_F_ (red) main chain atoms.

Figure 8 shows the 2D-NOESY NMR spectrum of M-*l*-Gln run at pH 4.5 in water added with 10% D_2_O, together with the significant cross-peak intensities. The strong NOE contacts between the topologically distant H_B_–H_D_ hydrogen atoms are indicative of main chain–side chain interactions. This structural information agrees with the simulated PDF of the H_B_–H_D_ distances (Figure 9). The sharp peaks centered at about 2.5, 4.0 and 4.8 Å (blue) are consistent with the close proximity of the side chain H_B_ hydrogens with the main chain H_D_ hydrogens, confirming the NOESY results. This spatial proximity and the presence of multiple sharp peaks of different intensities suggest that the interactions involve different repeat units at variable topological distances from the main chain, brought to proximity by the ordered coil conformation. Moreover, the broad weak red peaks in the range 2 < *r* < 10 Å (Figure 9) indicate weak interactions between the side chain H_B_ hydrogens and the main chain H_F_ hydrogens. This correlate with the weak NOE contacts found in the NOESY NMR spectrum (Figure 8). 

## 4. Conclusions

A new PAAC, M-*l*-Gln, was synthesized by the Michael polyaddition of *l*-glutamine with MBA, assuming that the *l*-glutamine moieties could form H-bonds through their lateral amide substituents. The M-*l*-Gln had higher molecular weight and higher hydrodynamic radius than all PAACs as determined by DLS and NMR diffusion studies. Aggregation mediated by intermolecular H-bonds could not be excluded. The acid–base properties of M-*l*-Gln were studied and the pH-dependent ionic speciation diagrams obtained. The CD spectra of M-*l*-Gln in water in the wavelength interval 200–280 nm showed pH-dependent, quickly achieved, and reversible self-structuring. Structuring was insensitive to ionic strength but sensitive to protein denaturing agents such as urea. 

Room temperature molecular dynamics simulations with final energy minimization using molecular mechanics methods were performed on the isolated M-*l*-Gln decamer as model. The optimized geometries in explicit water at pH 1.0, 4.5 and 12.0, revealed compact conformations characterized by parallel dispositions of topologically distant main chain strands in hairpin-like motifs. This behavior was in line with that previously observed for all chiral PAACs [32,33] and in contrast with the normal behavior of polyelectrolytes in water. The peculiar self-ordered coil conformations of M-*l*-Gln were stabilized by the establishment of H-bonds. These were particularly abundant at the isoelectric point, pH 4.5, mainly involving the main chain amide N–H and the side chain amide C=O. The PDFs, calculated during the MD run, confirmed the presence of intramolecular H-bonds between main chain and side chain groups belonging to topologically distant repeat units. Measurement using VT-NMR at pH 4.5 indicated that both main chain and side chain amide N–H were involved in H-bond formation. The 2D-NOESY NMR experiments at pH 4.5 evidenced a close proximity between main chain and side chain methylene groups, in agreement with the results of theoretical modeling.

As a final conclusion, the fact that M-*l*-Gln, like all other chiral PAACs, assumed a self-ordered hairpin conformation in spite of the structural diversity of their repeat units, confirmed that the propensity of PAACs for self-ordering in water solution was general and mainly induced by the arrangement of the main chain, in turn governed by the chiral carbon atoms of the α-amino acid moieties in a proximal position. 

## Figures and Tables

**Table 1:** Molecular weights of PAACs by SEC. ^a^

Sample	*Mw* ^b^	PDI ^c^
M-*l*-Gln	21500	2.31
M-*l*-Ala **^d^**	7800	1.44
M-*l*-Val **^d^**	5600	1.30
M-*l*-Leu **^d^**	6200	1.51

**^a^** Size exclusion chromatography. **^b^** *Mw* = weight average molecular weight. **^c^** Polydispersity index = *Mw/Mn*. **^d^** From Reference [33].

**Table 2:** *pK*_a_ and *β* values of M-*l*-Gln in the *α* range 0.2–0.8 (*β*_1_) and 0.1–0.9 (*β*_2_). ^**a**^

*pK*_a1_^b^	*pK*_a2_^c^	*β*_1_^b^	*β*_2_^c^	IP ^d^
2.17 ± 0.05	6.80 ± 0.05	0.84 ± 0.09	1.62 ± 0.08	4.5

**^a^** Averaged over four experiments. **^b^** Carboxyl group. **^c^** *Tert*-amine group. **^d^** Isoelectric point.

**Table 3:** Properties of the simulated M-*l*-Gln decamer at different pH values in water at the end of the MD runs and final geometry optimization.

pH	Charge	*R_g_*^a^ (nm)	*S*^b^ (nm^2^)	Volume ^c^ (nm^3^)	*μ*^d^ (D)
1.0	L^+^	1.07	25.1	2.91	19.8
4.5	L^0^	0.82	17.1	3.23	32.1
12.0	L^−^	0.98	23.5	2.96	23.2

^a^ Gyration radius. ^b^ Surface area accessible to the solvent. ^c^ Volume of the optimized molecule in water. ^d^ Dipole moment.

## Supplementary Materials

The following are available online at https://www.mdpi.com/2073-4360/12/4/881/s1, Figure S1: ^1^H-NMR spectrum of M-*l*-Gln recorded at pH 4.5 in: panel (a) 9:1 H_2_O:D_2_O and panel (b) D_2_O using a Brüker Avance III 400 MHz instrument. For the sake of clarity, the chemical shift assignments are also reported in Table S1. Figure S2: ^13^C-NMR spectrum of M-*l*-Gln recorded in D_2_O at pH 4.5 using a Brüker Avance 400 MHz instrument. For the sake of clarity, the chemical shift assignments are also reported in Table S1. Figure S3: ^1^H,^13^C-HSQC NMR spectrum of M-*l*-Gln recorded in 9:1 H_2_O:D_2_O at pH 4.5 using a Brüker Avance III 400 MHz instrument. Figure S4: Titration and speciation curves referred to the 1st experiment of Table S2 for M-*l*-Gln. Panel (a): experimental, simulated and *β* corrected titrations; panel (b): distribution of charged species. Determination of *β* parameters for –COOH and tert-amine of M-*l*-Gln referred to the 1st experiment of Table S2. Panel (c): calculation of *β* values from Equation (S1); panel (d): trends of the *β*-corrected *pK_a_* values versus α according to Equation (S1). Figure S5: Panel (a) NMR DOSY spectrum of M-*l*-Gln recorded in D_2_O at pH 4.5 using a Brüker Avance 600 MHz instrument: panel (b) linear fit of the logarithm of the intensity of H_D_ with respect to the square of the gradient strength. Figure S6: VT ^1^H-NMR spectra of M-*l*-Gln recorded in 9:1 H_2_O:D_2_O at pH 4.5 at 298, 318 and 338 K using a Brüker Avance 600 MHz instrument. Expansion of the amide N–H region, Table S1: Chemical shift assignments of ^1^H and ^13^C of M-*l*-Gln and the diffusion coefficients obtained by DOSY experiments. Table S2: *pK_a_* Values of M-*l*-Gln from different experiments.
