# Supplementary material for: Hydrogen Bonding in a l-Glutamine-Based Polyamidoamino Acid and its pH-Dependent Self-Ordered Coil Conformation
Source: Polymers (Basel). 2020 Apr 10;12(4):881. doi: 10.3390/polym12040881 (PMC7240574; doi:10.3390/polym12040881)
Supplement: Supplementary file 1 [file polymers-12-00881-s001.pdf]

Supplementary Materials

Hydrogen Bonding in a *L*-Glutamine-Based Polyamidoamino Acid and its pH-Dependent Self-Ordered Coil Conformation

Federica Lazzari [1], Amedea Manfredi [1], Jenny Alongi [1], Fabio Ganazzoli [2], Francesca Vasile [1], Giuseppina Raffaini [2]*, Paolo Ferruti [1]* and Elisabetta Ranucci [1]*

[1] Dipartimento di Chimica, Università degli Studi di Milano, via C. Golgi 19, 20133 Milano, Italy; federica.lazzari@unimi.it (F.L.); amedea.manfredi@unimi.it (A.M.); jenny.alongi@unimi.it (J.A.); francesca.vasile@unimi.it (F.V.)

[2] Dipartimento di Chimica, Materiali ed Ingegneria Chimica "G. Natta" Politecnico di Milano, piazza Leonardo da Vinci 32, 20131 Milano, Italy; fabio.ganazzoli@polimi.it (F.G.)

* Correspondence: giuseppina.raffaini@polimi.it (G. R.); paolo.ferruti@unimi.it (P.F.); elisabetta.ranucci@unimi.it (E.R.); Tel.: +39-02-23993068 (G.R.); Tel.: +39-02-50314128 (P.F.); +39-02-50314132 (E.R.)

Pages S1-S9

Figure S1-S6

Figure S1. ^{1}H-NMR spectrum of M-*L*-Gln recorded at pH 4.5 in: panel (a) 9:1 H_2O:D_2O and panel (b) D_2O using a Brüker Avance III 400 MHz instrument. For the sake of clarity, the chemical shift assignments are also reported in Table S1.

Figure S2. ^{13}C-NMR spectrum of M-*L*-Gln recorded in D_2O at pH 4.5 using a Brüker Avance 400 MHz instrument. For the sake of clarity, the chemical shift assignments are also reported in Table S1.

Figure S3. ^{1}H,^{13}C-HSQC NMR spectrum of M-*L*-Gln recorded in 9:1 H_2O:D_2O at pH 4.5 using a Brüker Avance III 400 MHz instrument.

Figure S4. Titration and speciation curves referred to the 1st experiment of Table S2 for M-*L*-Gln. Panel (a): experimental, simulated and β corrected titrations; panel (b): distribution of charged species. Determination of β parameters for -COOH and *tert*-amine of M-*L*-Gln referred to the 1st experiment of Table S2. Panel (c): calculation of β values from Equation (S1); panel (d): trends of the β-corrected pK_a values versus α according to Equation (S1).

Figure S5. Panel (a) NMR DOSY spectrum of M-*L*-Gln recorded in D_2O at pH 4.5 using a Brüker Avance 600 MHz instrument; panel (b) linear fit of the logarithm of the intensity of H_D with respect to the square of the gradient strength.

Figure S6. VT ^{1}H-NMR spectra of M-*L*-Gln recorded in 9:1 H_2O:D_2O at pH 4.5 at 298, 318 and 338 K using a Brüker Avance 600 MHz instrument. Expansion of the amide N-H region.

Tables S1-S2

Table S1. Chemical shift assignments of ^{1}H and ^{13}C of M-*L*-Gln and diffusion coefficients obtained by DOSY experiments.

Table S2. pK_a Values of M-*L*-Gln from different experiments.

References

NMR characterization

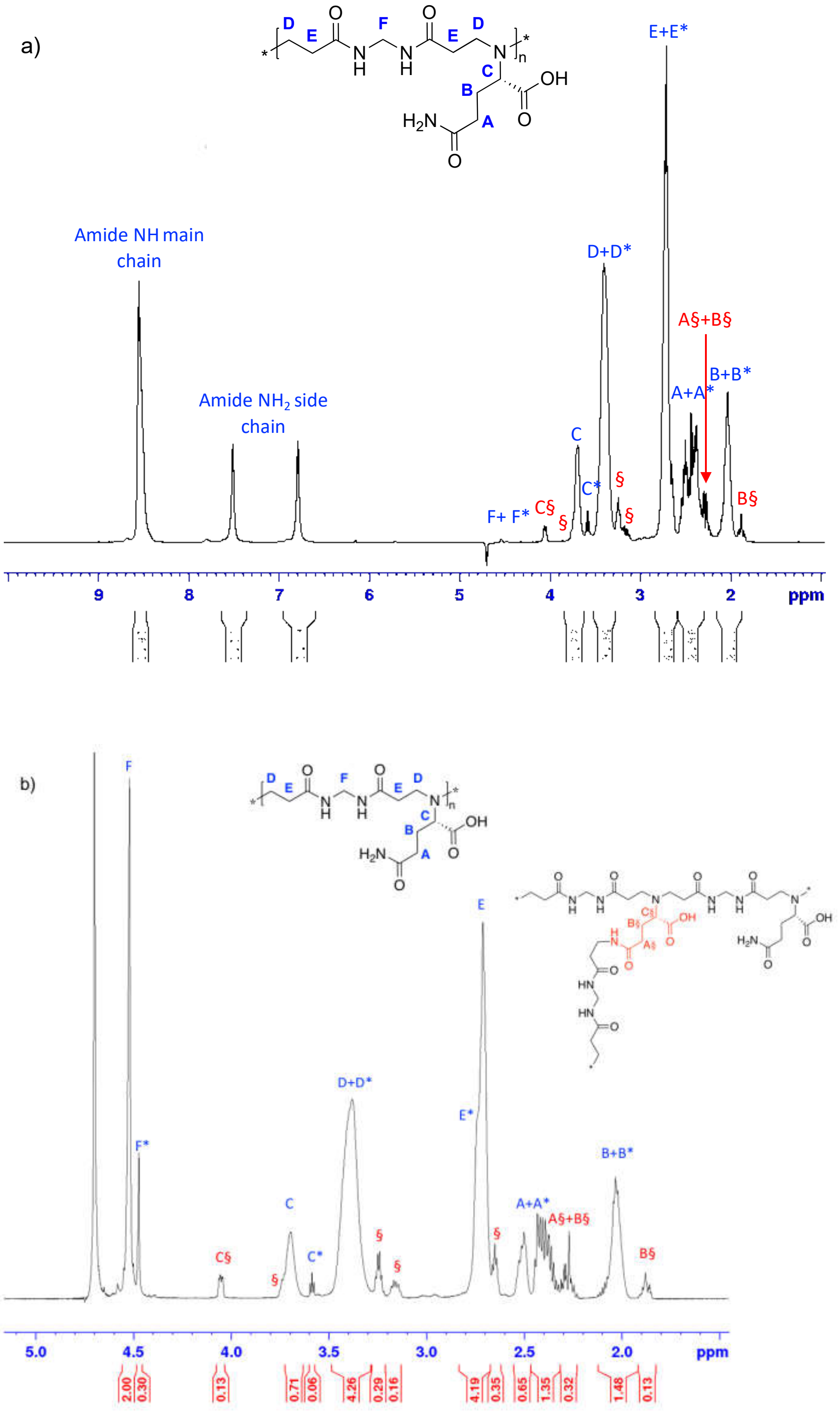

Figure S1. ^{1}H-NMR spectrum of M-*L*-Gln recorded at pH 4.5 in: panel (a) 9:1 H_2O:D_2O and panel (b) D_2O using a Brüker Avance III 400 MHz instrument. In the (1a) spectrum the solvent signal has been suppressed by excitation sculpting sequence. For the sake of clarity, the chemical shift assignments are also reported in Table S1.

* Terminals

§ Branches

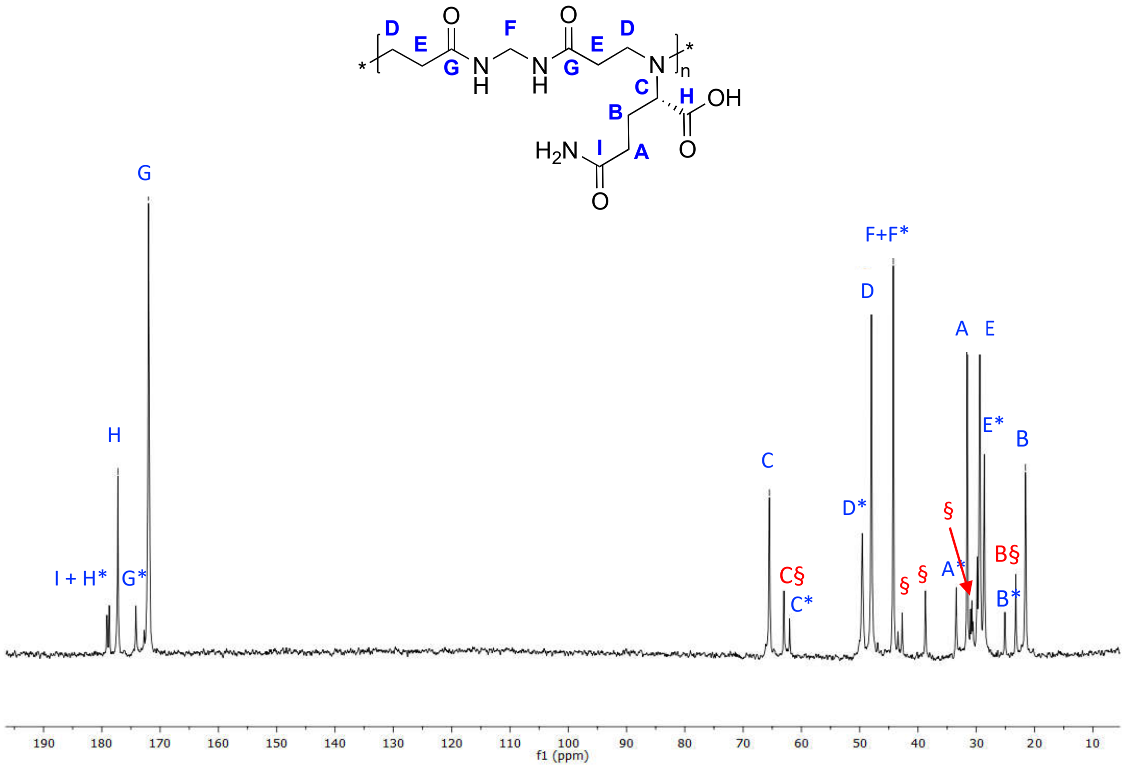

Figure S2. ^{13}C-NMR spectrum of M-*L*-Gln recorded in D_2O at pH 4.5 using a Brüker Avance 400 MHz instrument. For the sake of clarity, the chemical shift assignments are also reported in Table S1.

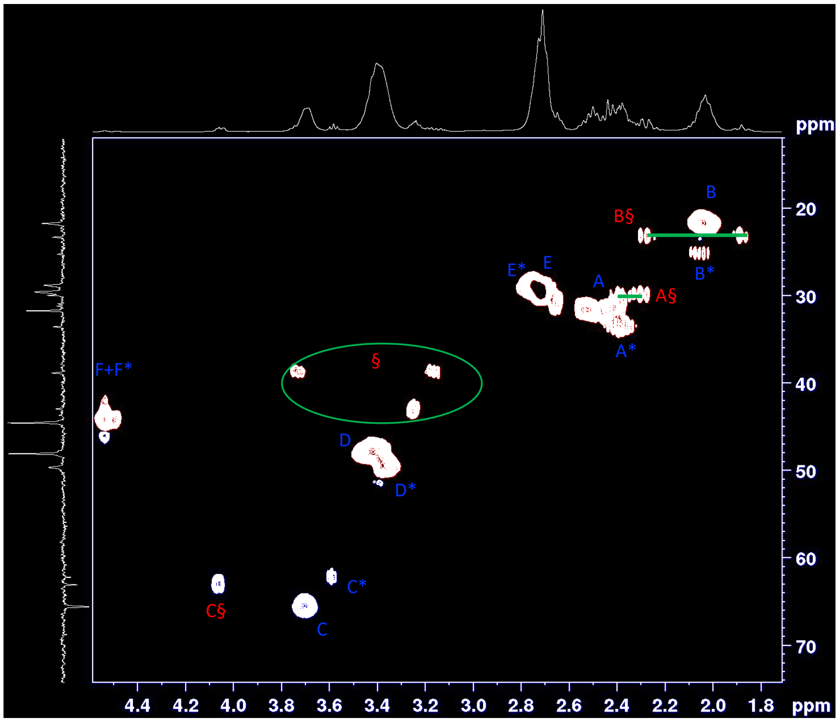

Figure S3. ^{1}H,^{13}C-HSQC NMR spectrum of M-*L*-Gln recorded in 9:1 H_2O:D_2O at pH 4.5 using a Brüker Avance III 400 MHz instrument. Color code: CH_2 red and CH blue.

Determination of pK_a and β parameter values and speciation curves

pK_a determination. The pK_{a1} (-COOH) and pK_{a2} (*tert*-amine) values of the ionizable functions of M-*L*-Gln were equivalent to the pH values at the half-equivalence points in the respective buffer zone of interest. The half-equivalence points were estimated as the pH values where half of the titrant volumes between consecutive inflections were added. The inflection points were in turn determined by numerically calculating the second derivative of the pH versus volume curves (Figure S4a).

β parameter determination. The β parameters of the generalized Henderson-Hasselbalch equation (Equation (1) in the manuscript, here reported as Equation (S1)) were determined, for both pK_{a1} (-COOH) and pK_{a2} (*tert*-amine), from Equation (S1) as the slope of the pH versus –log((1-α)/α) curve (Figure S4c). The points near inflections approached the validity limit of the logarithmic function and were not considered. Figure S4d shows the trends of the β-corrected pK_a values versus α according to Equation (S1).

$$pH = pK_{\mathrm{a}} - \beta \, log \frac{1-\alpha}{\alpha} \qquad \text{(S1)}$$

Simulation of the titration curves. Simulated titration curves were obtained following the De Levie approach [1] in order to iteratively refine pK_a and β values to achieve the best fitting of the experimental data.

- Initial conditions:

V_0 = initial solution volume
c_0 = initial PAACs concentration expressed as molarity of the repeat unit
c_S = initial concentration of ionic strength stabilizer
c_t = titrant concentration
V_t = volume of the titrant added
c_A = acid concentration used to correct pH
N = moles of strong acid possibly present as residual from the synthetic process or M-*L*-Gln pre-treatments

- *Mass balance:*

$$C_{M-L-Gln} = C_{L^+} + C_{L^0} + C_{L^-} = \frac{C_0 V_0}{V_0 + V_t} \qquad \text{(S2)}$$

$C_{M\text{-}L\text{-}Gln}$ refers to the repeat unit molar concentration.

- *Equilibrium constants* (S3a-S3c):

$$K_{a1} = \frac{C_{L^0} C_{H^+}}{C_{L^+}} \ \text{(S3a)}; \quad K_{a2} = \frac{C_{L^-} C_{H^+} y^2}{C_{L^0}} \ \text{(S3b)}; \ K_w = C_{H^+} C_{OH^-} y^2 \ \text{(S3c)};$$

- *Concentration fractions* (S4a-S4c):

$$\alpha_2 = \frac{C_{L^+}}{C} = \frac{C_{H^+}^2}{D} \ \text{(S4a)}; \qquad \alpha_1 = \frac{C_{L^0}}{C} = \frac{C_{H^+} y^2 K_{a1}}{D} \ \text{(S4b)}; \quad \alpha_0 = \frac{C_{L^-}}{C} = \frac{K_{a1} K_{a2}}{D} \ \text{(S4c)};$$

with:

$$D = C_{H^+}^2 + C_{H^+}K_{a1} + K_{a1}K_{a2} \qquad (S5)$$

The activity coefficients (Davies equation [2]):

$$y = 10^{-0.5\left[\frac{\sqrt{I}}{1+\sqrt{I}}-0.3I\right]} \qquad (S6)$$

Ionic strength:

$$I = \frac{1}{2}(C_{H^+} + C_{OH^-} + C_{Na^+} + C_{Cl^-} + C_{L^+} + C_{L^-}) \qquad (S7)$$

- <u>*Charge balance*</u>:

$$H^+ + Na^+ + L^+ = L^- + OH^- + Cl^- \quad (S8)$$

where (S9a-S9e):

$$C_{Na^+} = \frac{C_T V_T + C_S V_0}{V_0 + V_T} \ (S9a); \qquad C_{Cl^-} = \frac{C_S V_0 + C_A V_A + N}{V_0 + V_T} \ (S9b); \qquad C_{L^+} = \frac{\alpha_2 C_0 V_0}{V_0 + V_T} \ (S9c);$$

$$C_{L^-} = \frac{\alpha_0 C_0 V_0}{V_0 + V_T} \ (S9d); \qquad C_{OH^-} = \frac{K_w}{C_{H^+} y^2} \ (S9e);$$

Combining all former conditions, the following solving equation, representing the whole forward titration curve, was obtained in terms of V_T as a function of pH:

$$V_T = \frac{V_0[C_0(\alpha_0 - \alpha_2) + C_A - \Delta] + N}{\Delta + C_T} \qquad (S10)$$

where:

$$\Delta = H^+ - OH^- = H^+ - {K_w}/{H^+ y^2} \qquad (S11)$$

Simulated titration curves (Figure S4a) were obtained from Equations (S10) and (S11) by introducing the values of pK_{a1} and pK_{a2} in the respective buffer regions of interest, corrected for β_1 and β_2. Calculation were carried out considering C_{Na^+} and C_{Cl^-} constant throughout the whole titration experiment and equal to 0.1 M. Concentration fractions α and pK_a values were refined iteratively to achieve the best fitting to the experimental points.

Determination of speciation diagrams. Speciation diagrams (Figure S4b) were obtained by plotting the concentration fractions of the different ionic species as a function of pH (Equations (S12a-S12c)):

$$\alpha_2 = \frac{C_{L^-}}{C} = \frac{C_{H^+}^2}{D} \qquad \text{(S12a)}$$

$$\alpha_1 = \frac{C_{L^0}}{C} = \frac{C_{H^+} y^2 K_{a1}}{D} \qquad \text{(S12b)}$$

$$\alpha_0 = \frac{C_{L^-}}{C} = \frac{K_{a1} K_{a2}}{D} \qquad \text{(S12c)}$$

With D and y as previously described, and where the K_{a1} and K_{a2} values were corrected for β_1 and β_2.

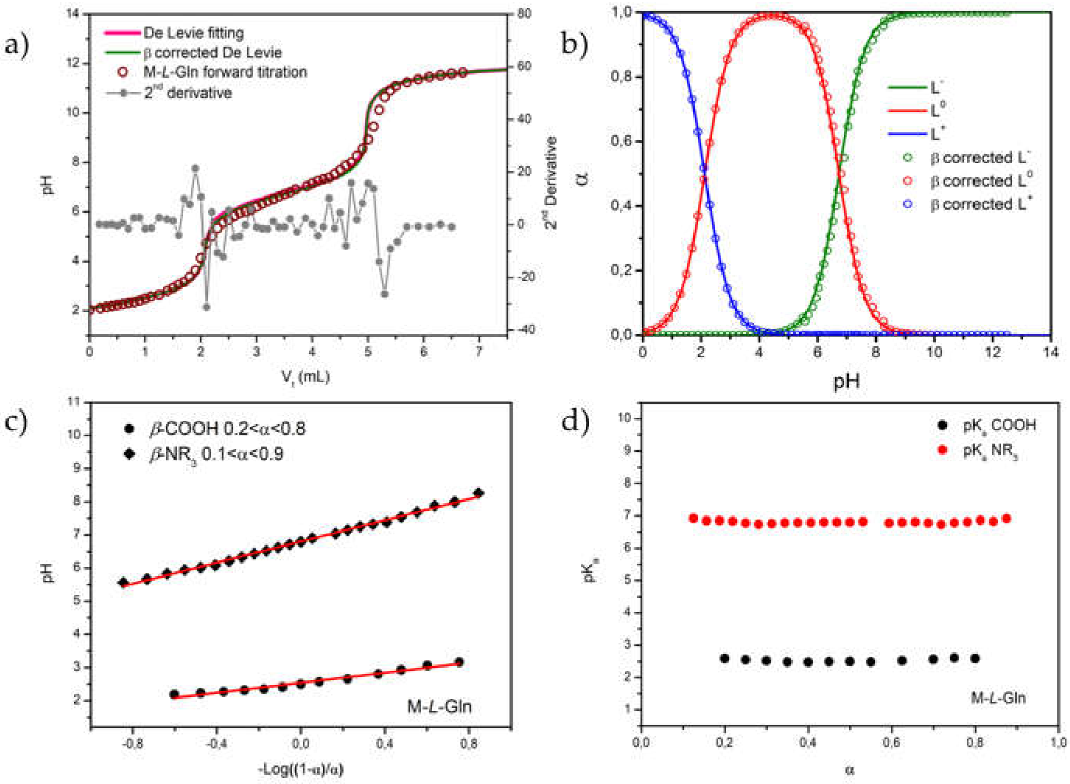

Figure S4. Titration and speciation curves referred to the 1st experiment of Table S2 for M-L-Gln. Panel (a): experimental, simulated and β corrected titrations; panel (b): distribution of charged species. Determination of β parameters for -COOH and *tert*-amine of M-L-Gln referred to the 1st experiment of Table S2; panel (c): calculation of β values from Equation (S1); panel (d): trends of the β-corrected pK_a values versus α according to Equation (S1).

Determination of diffusion coefficient, *D*, by DOSY experiments

DOSY spectra were recorded in D_2O at pH 4.5 using a Brüker Avance 600 MHz**,** following the standard Bruker sequence with pre-saturation during relaxation delay for water suppression. The diffusion coefficient, *D*, was determined from Equation (S13):

$$f(g) = I_0 \ e^{-\gamma^2 \cdot g^2 \cdot \delta^2 \cdot (\Delta - \delta/3) \cdot D} \qquad \text{(S13)}$$

where *f(g)* is the intensity as function of *g*, *g* the magnetic field gradient strength, I_0 the initial intensity, γ the gyromagnetic ratio 4.258 10^3 Hz/G, δ and Δ the delays, in particular δ the little delta value (2500 μs) and Δ the big delta value (200 ms), *D* the diffusion coefficient.
The *D* values for each proton are reported in Table S1.

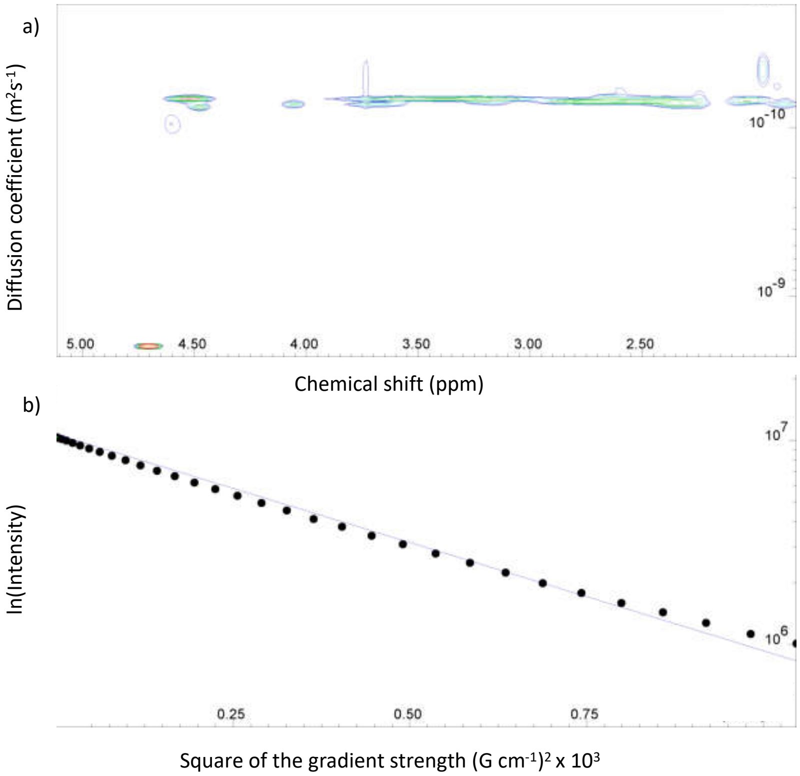

Figure S5. Panel (a) NMR DOSY spectrum of M-L-Gln recorded in D_2O at pH 4.5 using a Brüker Avance 600 MHz instrument; panel (b) linear fit of the logarithm of the intensity of H_D with respect to the square of the gradient strength.

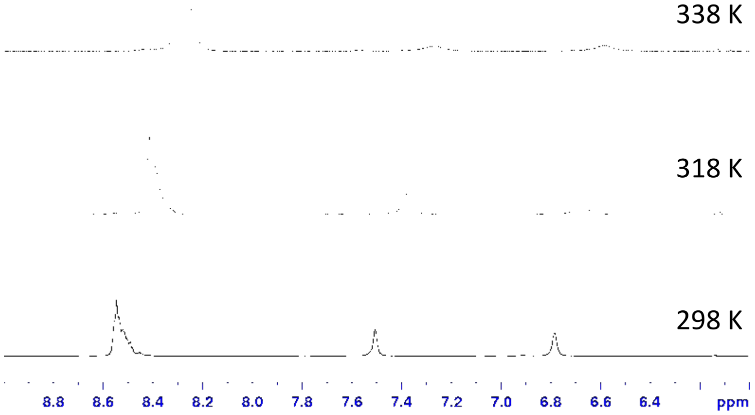

Figure S6. VT ^{1}H-NMR spectra of M-*L*-Gln recorded in 9:1 H_2O:D_2O at pH 4.5 at 298, 318 and 338 K using a Brüker Avance 600 MHz instrument. Expansion of the amide N-H region.

Table S1. Chemical shift assignments of ^{1}H and ^{13}C of M-*L*-Gln and diffusion coefficients obtained by DOSY experiments.

Hydrogen atom	^{1}H chemical shift (ppm)	Carbon atom	^{13}C chemical shift (ppm)	D (m^2s^{-1} x 10^{-11})
H_A	2.51-2.44	C_A	31.6	6.79
H_B	2.04	C_B	21.6	6.91
H_C	3.70	C_C	65.5	7.21
H_D	3.41	C_D	48.0	6.87
H_E	2.73	C_E	29.4	7.05
H_F	4.53	C_F	44.2	6.84
-	-	C_G	172.0	-
-	-	C_H	177.3	-
		C_I	178.5	
Amide NH_2 side chain	7.50 and 6.80			
Amide NH main chain	8.50			

Table S2. pK_a Values of M-*L*-Gln from different experiments.

Titration run	1st		2nd		3rd		4th	
	-COOH	$-NR_3$	-COOH	$-NR_3$	-COOH	$-NR_3$	-COOH	$-NR_3$
pK_a	2.21	6.79	2.11	6.76	2.21	6.87	2.15	6.76

References

1. De Levie, R. *How to Use ExcelW in Analytical Chemistry and in General Scientific Data Analysis*; Cambridge University Press: Cambridge, **2001**.

2. Davies, C. W. *Ion Association*. Butterworths: London, 37-53, **1962**.
